# Resilient Wireless Sensor Networks Using Topology Control: A Review

**DOI:** 10.3390/s151024735

**Published:** 2015-09-25

**Authors:** Yuanjiang Huang, José-Fernán Martínez, Juana Sendra, Lourdes López

**Affiliations:** Centro de Investigación en Tecnologías Software y Sistemas Multimedia para la Sostenibilidad (CITSEM), Campus Sur Universidad Politécnica de Madrid (UPM), Ctra. de Valencia, km. 7. 28031 Madrid, Spain; E-Mails: yuanjiang@diatel.upm.es (Y.H.); jsendra@etsist.upm.es (J.S.); lourdes.lopez@upm.es (L.L.)

**Keywords:** wireless sensor networks, fault-tolerant, resilience, self-healing, fault tolerance, topology control

## Abstract

Wireless sensor networks (WSNs) may be deployed in failure-prone environments, and WSNs nodes easily fail due to unreliable wireless connections, malicious attacks and resource-constrained features. Nevertheless, if WSNs can tolerate at most losing *k* − 1 nodes while the rest of nodes remain connected, the network is called *k* − *connected*. *k* is one of the most important indicators for WSNs’ self-healing capability. Following a WSN design flow, this paper surveys resilience issues from the topology control and multi-path routing point of view. This paper provides a discussion on transmission and failure models, which have an important impact on research results. Afterwards, this paper reviews theoretical results and representative topology control approaches to guarantee WSNs to be *k* − *connected* at three different network deployment stages: pre-deployment, post-deployment and re-deployment. Multi-path routing protocols are discussed, and many NP-complete or NP-hard problems regarding topology control are identified. The challenging open issues are discussed at the end. This paper can serve as a guideline to design resilient WSNs.

## 1. Introduction

Wireless sensor networks (WSNs) [1,2] have attracted much interest in recent years. The use of WSNs in numerous applications, such as forest monitoring, disaster management, space exploration, factory automation, secure installation, border protection and battlefield surveillance [3], is on the rise. WSN technology is the basis of the future network “Internet of Things” (IoT) [4,5]. The WSNs nodes are usually battery powered, deployed either randomly or according to a predefined statistical distribution [6] over a environment. Nodes are possibly prone to unexpected failures and malicious attacks. For example, in environment surveillance, the sensor node is exposed to an unsafe situation, making nodes likely to suffer various kinds of damage. On the other hand, the sensor node itself is very vulnerable due to the unreliable wireless connection and resource constrained features, such as limited transmission power, computing ability, storage space, *etc*.

### 1.1. Network Resilience

Network resilience is defined as the network ability to provide and maintain an acceptable level of service when facing various faults and challenges to its normal operation [7]. The work in [8] defines the resilience as the probability of at least having another path within the time interval *T*, given that at least one node on the primary path has failed. It is considered that resilience is not only the desirable requirement for WSNs, but also for any other network and control system. For instance, resilience is commonly found in natural systems and is a hallmark of the biological world [9]. Many analysts in engineering do not differentiate resilience from robustness and survivability [10]. Robustness and survivability tend to be used interchangeably with resilience, even though in practice, these properties are quite different [9]. Resilience, robustness and survivability are system-wide properties [11]. Resilience can also be described as the ability to recover to original status. The goal of resilience is to build fault-tolerant networks. By contrast, robustness stresses the ability to resist attacks, but does not imply an ability to restore from failure [9]. If the system is robust, that means that the system is very difficult to degrade under various attacks. As for survivability, this indicates that the system is very hard to impair completely.

In ecology, resilience can be measured by looking at the time that the system takes to return to its equilibrium state after a perturbation [11]. However, it is difficult to provide a clear quantitative assessment of network resilience to node failure [12]. The conventional evaluation is the number of failures the network can sustain before network disconnection or the probability that the network has to keep itself connected [13]. Briefly, if the network can tolerate at most *k* − 1 node failures and the resulting network is still connected, the network is called a *k* − *connected* network. If the network is *k* − *connected*, this also means that there are at least *k* − *disjoint* paths from any given two distinct nodes. Therefore, if one path fails, there are still *k* − 1 alternative paths available. On the other hand, the resilience of WSNs also depends on the link quality. Reliable links can make better connectivity quality.

### 1.2. Our Contributions

**Figure 1:**
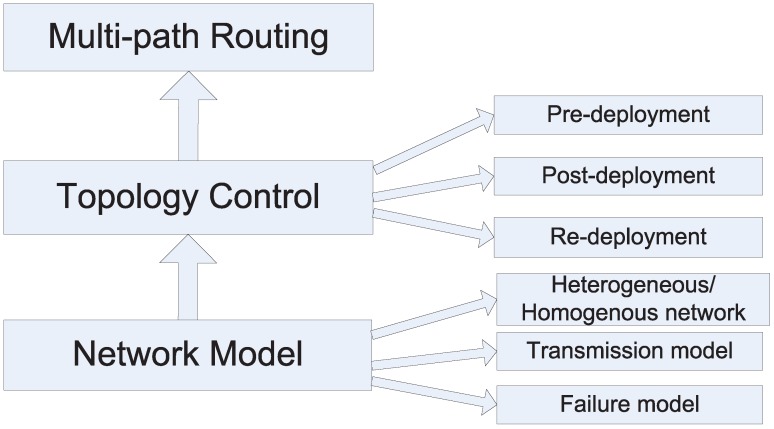
Resilient WSN design flow.

This paper focuses on surveying resilient design in WSNs from the topology control and routing protocol perspective. Figure 1 shows the main flow of designing resilient WSNs. As a matter of fact, there are many other ways to achieve resilience. In the layered network model, the physical, link, network, transport and application layer can have their own resilient strategies. For instance, redundant coding allows detecting and correcting coding error; multi-processor systems have better fault tolerance performance; the security mechanisms protect data confidentiality, integrity and availability, *etc*., but those methods are out of the scope of this paper. More specifically, this paper surveys techniques on how to build a *k* − *connected* network, where *k* is the most important indicator for resilience, and how to find out the alternative paths by multi-path routing protocols. In general, the resilience capability of WSNs relies on the redundant deployment and adaptive design. Thanks to the low cost of WSNs nodes, unlike traditional networks, it is possible to deploy far more nodes than what is actually needed to make the network fault tolerant. Researchers, implicitly or explicitly, leverage a network model; hence, this paper first discusses the network model in WSNs. It is shown that most works employ inappropriate network models, and it is complicated to comprehensively model real WSNs. Afterwards, this paper reviews the theoretical results and representative topology control approaches to guarantee WSNs to be *k* − *connected* in three different network deployment stages.

(1) pre-deployment stage: the literature focuses on either how many nodes are needed if the nodes are randomly deployed or how to construct a particular network topology if nodes are able to control their positions;

(2) post-deployment stage: the main focus is to maintain or to improve the node connectivity or link quality by adjusting the transmission range or transmission power;

(3) re-deployment stage: this aims at how to populate new nodes to recover from the network disconnection.

Usually, network resilience is not the only concern; other problems, such as network coverage, node energy or the number of nodes deployed, are also considered in the literature, which will be involved in the discussion.

Considering that a multi-path routing protocol is important for resilient WSNs and also one of the most active research areas in WSNs, this work also involves this part. Note that the network topology is the foundation of routing protocols. Routing algorithms can find alternative paths only because multiple paths already exist from the network topology perspective. Unfortunately, most problems related to topology control in WSNs have been proven to be either NP-complete or NP-hard, which is very difficult to solve. Therefore, those problems are identified in this work, as well. This paper would be useful for those researchers who want to design resilient WSNs from both a theoretical study and a practical application point of view.

This paper is organized as follows: Section 2 introduces the main terminologies and definitions that will be used in this paper, and Section 3 presents various network models and failure models. In Section 4, an extensive survey on topology control approaches is given in which they are categorized according to three deployment stages: pre-deployment stage, post-deployment stage and re-deployment stage. Multi-path routing is introduced in Section 5. Those NP-complete or NP-hard problems in WSNs are summarized in Section 6. The open issues are discussed in Section 7, and Section 8 concludes our work.

## 2. Resilient WSNs: *k-Connected* Network

From the network topology point of view, if the network is *k* − *connected*, the higher the *k* value, the more network resilience. WSNs can be modeled as simple undirected (or directed) graphs. This section briefly introduces the terminology and definitions that will be used in this paper and presents significant findings related to *k* − *connected* networks. For more graph terminologies, readers are referred to the book [14]. Throughout this paper, the terms “network” and “graph” are used interchangeably.

### 2.1. Terminology

**Figure 2:**
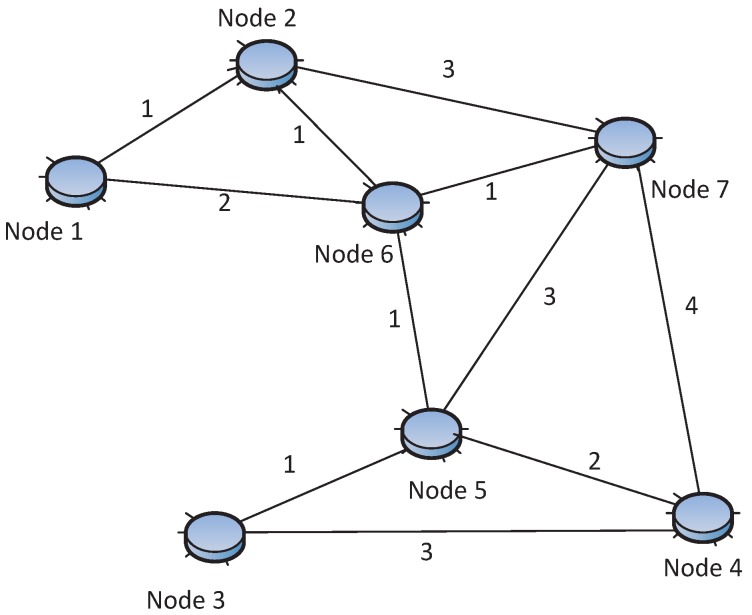
WSN topology example. (1) |*V*| = 7, |*E*| = 11; (2) This is the non-negative weighted edge undirected connected graph; (3) Four nodes, 6, 3, 7 and 4, are neighbors of Node 5, so *d*(*v*_5_) = 4; (4) Network degree *δ* = 2; (5) This is two-connected and two-edge-connected.

Formally, a WSN can be described as a simple, non-negative weighted edge undirected (or directed) graph *G* = (*V*, *E*) in a two-dimensional area, where *V* and *E* are the set of vertexes and edges, representing sensor nodes and links respectively, as shown in Figure 2. Each edge has a non-negative value called the weight, representing the cost of the edge, e.g., energy, distance, delay, *etc*. The cardinality of the vertex (sensor node) and edge (link) in the graph is denoted by |*E*| and |*V*|. *r_c,i_* denotes the communication range, and *r_s,i_* is the sensing range of vertex *i*. *r_c,i_* is the longest distance of the radio that a sensor can reach, and *r_s,i_* is the longest distance that a sensor can sense environmental phenomena (e.g., temperature), usually *r_c,i_* ≥ *r_s,i_*. The sensing and communication range of sensors are represented by disks, including their boundaries. The communication range and sensing range can be simply written as *r_c_* and *r_s_* in the case that all vertexes have the same communication and sensing range. For any two distinct vertexes *v_i_*, *v_j_* ∈ *V*, the vertex *v_i_* can connect to *v_j_* if and only if the Euclidean distance |*v_i_* − *v_j_*| ≤ *r_c,i_*. The neighbor set of *v_i_* is denoted as *N*(*v_i_*) and defined as any nodes within communication range of *v_i_*, namely *N*(*v_i_*) = {*v_j_* : |*v_i_* − *v_j_*| ≤ *r_c,i_*, *v_j_* ∈ *V*}. The number of neighbors of *v_i_* is called the degree of *v_i_*, denoted as *d*(*v_i_*). The graph degree is defined as *δ* = *min*(*d*(*v_i_*)) for all *v_i_* ∈ *V*. For example, in Figure 2, Node 5 has degree four and the graph degree is two. A point *p* is covered by the sensor node *v* only when the Euclidean distance is less than the sensing range, that is |*p* − *v*| ≤ *r_s,v_*.

In fact, the communication and sensing ranges are not necessarily a disk and in reality are not the hard boundary, e.g., the sensing range could be defined as the threshold of a false alarm rate. Therefore, the statistical nature of sensor network applications and the environments can be incorporated into the definitions of sensing range and communication range, e.g., [15]. This will be discussed in the network model section.

### 2.2. k − Connected and k − Coverage Network

In this section, the definitions of network connectivity and coverage are introduced.

**Definition 1.** *Connected graph: For any two distinct vertexes, if they can communicate with each other either directly or via a limited number of intermediate vertexes, then the graph is called a connected graph.*

**Definition 2.** *k-connected graph: Given integer k* ≥ 1*, if the graph is still connected after removing no more than k* − 1 *vertexes, then G is called k−vertex−connectivity graph, or a k−vertex−connected graph, usually simply denoted as a k − connectivity graph or k − connected graph. The value k is called connectivity. The vertex connectivity κ of the graph is the maximum k, such that the graph is a k − connected graph.*

**Definition 3.** *k-edge-connected: Given integer k* ≥ 1*, the graph is still connected after removing no more than k* − 1 *edges; G is named the k − edge − connectivity graph, or k − edge − connected graph. The vertex connectivity κ′ of the graph is the maximum k, such that the graph is a k − edge − connected graph.*

For example, in Figure 2, if two, Nodes 2 and 6, are removed, the resulting network is disconnected because Node 1 will be isolated; at the same time, removing only one node is unable to disconnect the network. The relation between vertex and edge connectivity is given by the following well-known Equation (1), which is called Menger’s theorem [14,16]:

(1) $$\kappa \leq \kappa^{\prime }\leq \delta \leq \frac{2|E|}{\left|V\right|}$$

Apparently, the connectivity *k* helps to evaluate the WSNs’ fault tolerance capability. The higher *κ* or *κ*′ is, the more resilient the WSNs will be. The upper bound of connectivity *κ* can be achieved by Harary graphs. The lower bound of *κ* is zero or $\left|E\right|-\frac{\left(|V|-1)(|V|-2\right)}{2}$ [16].

On the one hand, the connectivity in most cases is a desirable feature. A *k* − *connected* network is important for the key distribution problem in WSNs. For instance, [17,18,19,20,21,22,23,24] analyze the probability on the reliable connectivity of WSNs under the random key distribution scheme. More specifically, they study the conditions for how to scale the model parameters in a random key pre-distribution scheme called Eschenauer–Gligor (EG), so that the random key graph is *k* − *connected*. They show the correlation between the probability of being *k* − *connected* and the parameters in the EG scheme. The probability that a random key graph has a minimum node degree no less than *k*, but that is not *k* − *connected* converges to zero as 0 → ∞ [3]. The transmission model used in these works is Erdös–Rényi, rather than a disk model. A high degree also helps to detect failures. The work in [25] shows that optimal error detection decreases exponentially with the increase of degree. On the other hand, in a few cases, it is in fact an unwanted feature, e.g., virus propagation [26].

To some extent, improving network resilience means increasing, maintaining or constructing the required *κ* or *κ*′. In this paper, when we mention that the network is *k* − *connected*, this means *κ* − *connected*; if the network is *k* − *edge* − *connected*, this means *κ*′ − *edge* − *connected*.

**Definition 4.** *k-coverage network: the network is called a k − coverage network where k is the biggest positive integer, such that any point in the deployment area is covered by at least k distinct sensor nodes via sensing range.*

### 2.3. Partially-Connected Network

The definitions above require that the entire network is *k* − *connected* or *k* − *coverage*. However, as the WSNs are characterized by high density, only a small number of nodes is needed for connectivity or coverage in order to save energy and prolong the WSNs’ lifetime. As a result, maintaining a whole network as connected is not only quite difficult, but also maybe unnecessary. If the goal is not to maintain the whole network as connected, it is called a *partially connected network*.

**Definition 5.** *Partial k−connected network: [27,28] propose a concept called a partial k−connected network. If the original network is damaged, new nodes are added to the network. The goal is not to recover the entire new network as k − connected; instead, it only maintains the original nodes that are k − connected.*

Being partially-*k* − *connected* weakens the final objective, but is still useful in the case that only original nodes serve an important purpose, while the added nodes are only used to maintain connectivity. On the other hand, WSNs have many redundant nodes, so maybe only part of them is required to be connected, e.g., the giant component to be connected being as large as possible.

**Definition 6.** *Giant component threshold: there exists a critical value, such that whenever the fraction of removed nodes (or links, depending on the context) is lower than P_c_, the network still holds a giant component almost certainly; whereas, whenever the fraction of removed nodes (or links) is greater than P_c_, the network is almost sure not to have a giant component anymore.*

For random failures in a large random deployed network, *P_c_* is given by Equation (2) [29]:

(2) $$P_{c}=1-\frac{<k>}{<k^{2}>-<k>}$$

where $<k^{j}>=\sum_{k=0}^{\infty }k^{j}p_{k}$ (where *j* = 1, 2) and *p_k_* is the probability distribution for all *k*, e.g., Poisson distribution $p_{k}=e^{-z}\frac{z^{k}}{k!}$. Notice that this conclusion is suitable for any degree distribution, e.g., Poisson distribution, continuous and discrete power law. More details about the results under other two conditions are available in [29]: the number of nodes tends to infinity and a given large number *N*.

In WSNs, only the most important nodes are needed to be connected together sometimes. This theory is associated with the concept known as the minimal connected dominating set (CDS).

**Definition 7.** *Minimal connected dominating set (CDS): the connected dominating set D is a sub-graph of the graph G, such that: (1) D is connected to the sub-graph of G; (2) any vertex in G either belongs to D or is adjacent to a vertex of D. One graph may have more than one D. The one that has the minimal number of vertexes lower than any other connected dominating set is called the minimal CDS.*

Whether there is a connected dominating set with a size less than a given value is an NP-complete problem. So far, the best approximation of finding the minimal dominating set is a *O*(log |*V*|)-approximation [30] and a *O*(log Δ)-approximation distributed algorithm [31], where Δ is the maximal degree of the graph. There are several papers, such as [32,33], that present the topology control algorithms through constructing an optimal-sized CDS to achieve a trade-off between the network lifetime and the network coverage in WSNs.

## 3. Network Model

This section introduces the general network model related to WSNs. The network models are quite important to analyze WSNs; different network models may lead to different conclusions.

### 3.1. Heterogeneous and Homogenous Network

In a homogenous network, all nodes have the same performance in terms of communication range, power level, processing ability, mobility, memory, *etc*. Otherwise, it is called a heterogeneous network. Sometimes, in order to simplify the problem, one aspect is stressed, and others are ignored. For example, if each node has the same range, the network is homogenous; otherwise, it is heterogeneous. In practice, WSNs are very likely to be heterogeneous. For instance, the base station and cluster heads are more powerful than other nodes; hence, they have a longer communication range than regular nodes; especially, the sink node has no constraints with respect to energy, as it is usually not a battery-powered device.

### 3.2. Transmission Model

Usually, researchers employ ideal transmission models for WSNs, e.g., the node transmission model and sensing model are disks, the links between nodes are symmetric and the transmission radio is omnidirectional; unfortunately, the real transmission model is much more complicated.

#### 3.2.1. Irregular Radio Model

**Figure 3:**
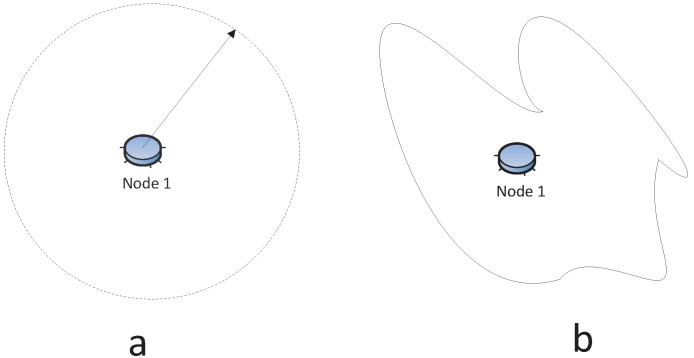
(**a**) Regular radio model; (**b**) Irregular radio model.

The sensor transmission is usually modeled as a disk centered at the node with its radius being its communication range, as shown in Figure 3a. However, the reception signal power attenuation relies on *R^−α^*, where *R* is the transmission range and *α* is the loss constant that depends on the wireless medium with typical values between two and four, and it can vary from device to device [34,35]. In fact, it has been found that the radio communication range is highly probabilistic and irregular [36,37], especially in the indoor environment [38], like Figure 3b. The works in [37,39] make it evident that the radio is irregular, even time-varying while stationary, in the MICA2 sensor platform. Most simulators do not have irregular radio models, because of the complexity of analyzing irregular radio models. Our previous study [40] proposes a probabilistic approach to describe the connection between nodes, since the radio model is irregular. More specifically, the network connection probability is modeled as a Cox process, and the numeric simulations are performed to evaluate the network connection probability.

It is worth noticing that [34] states that the WSNs cannot simply be modeled as a geometric graph where each node connects other nodes within its communication range. For wireless networks, only the maximum outgoing edge contributes to the energy consumption, but other nodes with a shorter communication range can be connected more or less for free, which makes studying energy-related issues in WSNs quite different. A wired network is a link-based or edge-based metric, while a wireless network is a node-based metric [34].

#### 3.2.2. Asymmetric Link

**Figure 4:**
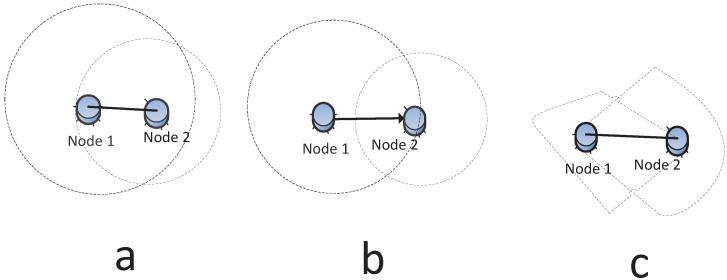
(**a**) Symmetric link: Node 1 can communicate with Node 2 and *vice versa*; (**b**) asymmetric link: Node 1 can send data to Node 2, but Node 2 cannot send data to Node 1; (**c**) non-omnidirectional radio.

A symmetric link implies that if one node is able to receive a message from another node, then it can send a message to the same node. For a wired network, this is true, but for a wireless network, this is true only when the communication range of both nodes is the same or the distance between two nodes is less than the minimum communication range of both nodes. The latter is called a mutual inclusion graph (MG) in [41]. However, in any real case, the network is likely to be asymmetric, so the resulting network is directed. The irregular communication range leads to an asymmetric link. Figure 4a,b shows the symmetric and asymmetric communication model, respectively.

The omnidirectional radio indicates that the node’s antenna broadcasts in all directions. In WSNs, it is possible that radio communications can be modeled as a directional antenna, which can transmit in specific direction(s), as illustrated in Figure 4c. The work in [42] suggests that both unreliable and asymmetric links have a great impact on the global quality of connectivity in large-scale sensor networks.

### 3.3. Failure Model

Communications over wireless channels are more insecure and susceptible to various attacks when compared to wired networks, e.g., the eavesdropping is easier on the wireless link. There are many reasons that cause network node(s) or link(s) failure. The failures may be caused by physical damage, such as fire, animal or vehicular accidents [27], or disasters, like deluges, earthquakes, sandstorms, *etc*. In the network, the failure can be modeled as the removal of nodes or links, or as the probability of nodes (links) failing. The difference is that when the node is removed, the corresponding links connected to it must be removed, as well. The work in [43] considers the statistically-independent and -dependent link failures under low stress (probability lower than 0.2) and high stress (probability more than 0.5) conditions, but without considering the node failure. The node failure model and link failure model seem quite different. However, [29] shows that the link failures are not qualitatively different from what is observed from the node point of view.

The failure model is very important and has significant influence on the algorithm design. It is possible that one algorithm is only suitable for a specific failure, e.g., a single node failed at one time. In practice, the malfunctioning of a single component of a real system can generate a cascading effect. The work in [8] considers two failure types: (1) isolated failures, where each node in the multi-path has a probability of failure during some small intervals *T*; and (2) patterned failures, the failure of all nodes in a circle within a given small time interval *T*, which is Poisson distributed. The isolated failure models the independent node failure, and patterned failure models the geographically correlated failure. The works in [44,45,46] consider simultaneous failures. Nevertheless, losing a large number of nodes simultaneously is a low probability event [36].

In this paper, the failures are classified into permanent failures, transient failures and faulty readings. To detect the node or link failure, the most common approach is broadcasting the messages and waiting for the response in a certain time interval in order to test the connectivity to other nodes. For example, in routing protocol Octopus [47], the package is broadcast by each node every hello timeout period. If a node does not hear from a neighbor for more than two times the hello timeout, it removes this neighbor from its neighbor list. Failure node detection is not the focus of this paper. A survey on failure detection in WSNs is given in [48].

#### 3.3.1. Permanent Failure Model

In the permanent failure model, the nodes fail forever and have no chance of recovering. The permanent failure model can be further categorized into: (1) random failures, where the failure occurs randomly in the WSNs; and (2) attacks, where the failures are due to deliberate damage, such as preferentially targeting most connected nodes. Random damage can be modeled as randomly removed nodes or links, but attacks are modeled by removing nodes or links following some strategy, for instance nodes may have a higher degree [29,49]. The work in [50] categorizes the attacks based on their impacts, including data integrity and confidentiality, power consumption, routing, identity, privacy and service availability.

The work in [13] analyzes that the single failure probability dominates the overall disconnection probability in a regular graph where each node has the same degree. In a random network with a size tending to infinity and the degree distribution being *p_k_*, after the removal of a fraction *p* of the nodes during a classical attack, which removes the highest degree node, the maximal degree *K*(*p*) is given by Equation (3) [29]:

(3) $$p=1-\sum_{k=0}^{K(p)}p_{k}$$

#### 3.3.2. Transient Failures Model

In the transient failure model, the failure occurs, but the system reverts to its former status after a time interval, or fluctuates between normal and abnormal status. The work in [47] takes into account when the network is intermittently disconnected and connected. Each time an unstable node awakens, it remains connected for a time interval chosen uniformly at random in the range [0, 120]. When it is disconnected, it remains disconnected for a time interval chosen uniformly at random in the range [0, 60].

#### 3.3.3. Faulty Reading

Faulty readings can be conceived of as the time when the node operates well although suffering from faulty readings. The sensors with faulty readings are called faulty sensors. These faults can be attributed to: (i) faulty data measurement or data collection; (ii) some variables in the area surrounding the sensor have changed significantly; or (iii) the inherent performance of the sensor is abnormal [51]. The faulty data can only be referred to as a deviation from the expected model [52]. More types of definitions of the faulty data can be found in [52]. To detect faulty nodes, the stochastic method is usually employed. For instance, given a defined threshold, the value it has is compared to the values of its neighbors, and the median value to filter out the faulty measurement is used, e.g., [25,51,53,54].

## 4. Topology Control

From the network topology point of view, the resilient WSN problem is a *k* − *connected* problem. The topology control is a common way to preserve or improve the connectivity of WSNs by controlling the network topology. Note that the connectivity is one of the most important, but usually not the only concern in WSNs’ design. Some other parameters, such as coverage, energy and number of nodes required, also should be taken into account to optimize. In most cases, there is a need to consider both the network coverage and connectivity, e.g., [15,36,55,56]. Some works survey the coverage problem in WSNs, e.g., [57,58].

In general, network connectivity is more important than other performances because collecting environment data is usually the primary task. The coverage and energy problems become less important if the sensor nodes are not able to forward the collected data to the destination.

### 4.1. Topology Control in Different Deployment Stages

**Figure 5:**
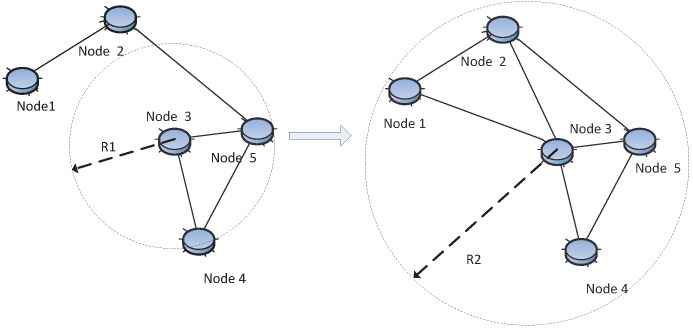
Topology control: adjust the transmission range of Node 3 from R1to R2 in order to improve connectivity.

**Figure 6:**
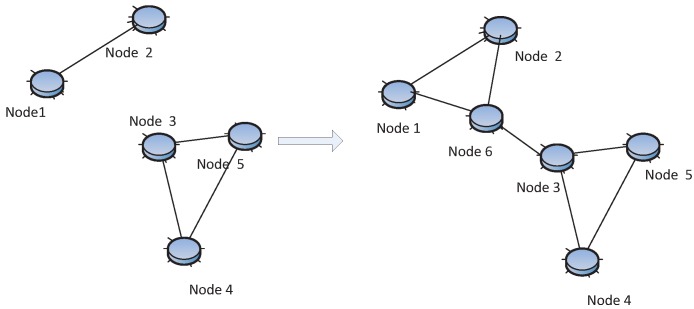
Topology control: the new Node 6 is added to recover the connection.

There are three deployment stages for WSNs: pre-deployment, post-deployment and re-deployment. For each stage, there are corresponding approaches to preserve, improve or recover network connectivity:

(1) pre-deployment stage, that is the sensor node positions are not fixed yet, and the network is not operational, e.g., it has not started sensing the environment. At this stage, deploying sensor nodes randomly or constructing a particular network topology to satisfy the required connectivity is the expected action to be taken.

(2) post-deployment stage, that is the sensor node positions are already known. At this stage, adjusting the transmitting range or equally node transmitting power to achieve desired connectivity is what is usually done The goal is to improve the connectivity while consuming as little energy as possible or to improve the link reliability, as shown in Figure 5.

(3) re-deployment stage, that is new nodes can be added to the original network. At this stage, new nodes are populated in the original network to maintain or repair the network connectivity, as shown in Figure 6. Usually, the goal is to improve the network connectivity with relatively less nodes.

Normally, the connectivity improvement strategies apply at only one stage. Unfortunately, all of them are challenging tasks, because most of them are either NP-complete or NP-hard. In Section 6, this paper lists many problems regarding network connectivity.

### 4.2. Pre-Deployment

Usually, there are two cases in the pre-deployment stage: nodes are randomly deployed or their locations can be controlled.

#### 4.2.1. Random Deployment

If the sensor nodes are randomly deployed, it is commonly assumed that the node position has a uniform distribution. The question is how many neighbors (or node degrees) a node needs so that the whole network will be *k* − *connected*. In the uniform distribution network, [59] states that six to 10 neighbors are able to make sure that the network is connected with high probability. The work in [60] concludes that the “lower bound” to make a network disconnected and the “upper bound” to make a network connected are 0.074 log *n* and 5.1774 log *n*, respectively, where *n* is the total number of nodes. The work in [61] observes that the upper bound is *αe* log *n*, where *α* > 1, *e* is the naturalbase. The work in [62] shows that if *k* ≤ 0.3043 log *n*, then the graph is not connected with a high probability, and if *k* ≥ 0.5193 log *n*, the network is connected with a high probability as *n* → +∞. The *k* − *connected* network is asymptotically the same as that for 1−*connected* [63], which indicates that once the network is 1−*connected*, it achieves *k* −*connected* immediately in the uniform distribution network. However, those asymptotical conclusions hold theoretically only when *n* → +∞, which is not so practical.

Our previous study [40] leverages a probabilistic approach to analyze the network connection probability and proves that if the probability of the network being connected is 0.36*ε*, then the probability of the network being connected is at least 1 − *ε* by means of increasing the communication range by constant *C*(*ε*), where 0 < *ε* < *e*^−1^. Explicit function *C*(*ε*) is found. Furthermore, the localized control algorithms based on fuzzy logic are proposed in our paper [46,64] to achieve the desired node degree. The simulation results show that the average node degree can be achieved in dynamic WSNs due to the closed-loop feedback of the control system. The proposal in [46] improves the fault-tolerate performance in the presence of random attacks. By combining the theory in [40] with the control algorithms in [46,64], a network being *k*−*connected* with a high probability can be designed, which will tolerate at most *k*−1 node failures.

If the nodes are uniformly and randomly deployed, the degree distribution follows the Poisson distribution, but in real life, degree distributions are strongly non-Poisson distribution, often taking the power law, truncated power-law or exponential forms [65]. The work in [66] shows that the degree of nodes in technological networks, such as the World Wide Web, the Internet and airplane connection networks, is a power law distribution. Some biological systems, such as metabolic networks and protein networks, are different from the uniform distribution networks and have a power law degree distribution with an exponent that ranges between two and three. The power law degree distribution is more vulnerable to deliberate attack than the Poisson degree distribution, but the power law degree distribution is more resilient to random failure than the Poisson degree distribution [65,66,67]. The reason is straightforward. For the uniform distribution networks, each node contributes equally to the network, so randomly removing nodes causes the same amount of damage. By contrast, for the non-uniform distribution network, each node plays a different role and attacks the most connected nodes (namely higher degree) causing heavy damage. The work in [68] shows a real case of an earthquake and tsunami that happened in Japan. In this case, the Internet was impressively resilient to the disaster due to its heterogeneity. However, [29] finds that when the number of nodes is a limited value, the difference between Poisson and power law networks in practice is not that significant; instead, it is overestimated. The network is more vulnerable if the network scale increases, but the degree of a regular network is constant, which implies that a large-scale network is more susceptible than a small-scale network if they both have the same degree. We believe that, like other networks, WSNs also are resilient to random failures, but vulnerable to attacks.

#### 4.2.2. Construct *k* − *Connected* Networks

When the nodes in the WSNs are able to control the locations, the questions are how to construct a *k* − *connected* network or how to verify that the constructed network is *k* − *connected*. At the same time, it is necessary to consider the *k* − *coverage* issue in some cases.

(1) Construct *k* − *Connected* and *k* − *Coverage* Network:

There is a well-known result showing that if the communication range is at least two times longer than the sensing range, that is *s_c_* ≥ 2*s_s_*, then *k* − *coverage* can guarantee *k* − *connected* [15]. If the node’s location is carefully designed, the ratio can be further optimized. For example, [69] proves that strip-based is the asymptotically optimal deployment for achieving both 1 − *coverage* and 1 − *connected* for all $\frac{r_{s}}{r_{c}}\leq \sqrt{3}$, and [70] proposes that a diamond pattern can achieve the asymptotic optimality for 1 − *coverage* and 4 − *connected* when $\frac{r_{s}}{r_{c}}>\sqrt{2}$. For higher connectivity, [71] presents the hexagon-based deployment pattern to achieve 1 ≤ *k* ≤ 6 connectivity. However, the optimal ratio between communication range and sensing range such that the network is *k* − *coverage* and *k* − *connected* for general *k* is still an open issue.

The work in [55] first shows the conditions that a network is *k* − *coverage* and *k* − *connected*. It states that the network is *k* − *coverage* and *k* − *connected* if it is *k* − *DPC*. *k* − *DPC* is defined as: if each point on the perimeter of every node is covered by at least *k* other nodes through the sensing range, each node has a link to all of its neighbors. Here, two nodes being neighbors means that there exists an intersection between their sensor ranges, rather than the communication ranges. The distributed protocols are proposed to guarantee *k* −*coverage* and *k* −*connected* under the condition that the initial coverage and connectivity are higher than expected. Similarly, [72] also proves that the target field is *k* − *coverage* only when the sensing border of every node is *k* − *coverage*. It uses this conclusion to verify whether the network is *k*−*coverage* or a coverage hole exists in a *d*−*dimension* network, where *d* is 1, 2, 3.

On the contrary, given the ratio between *r_c_* and *r_s_*, the connectivity can be calculated. The work in [36] suggests that if communication range *r_c_* is at least equal to sensing range *r_s_* and the network is connected, its connectivity *k* is $\frac{2k\pi r_{c}^{2}}{\left(\pi -\sqrt{3}\right)r_{s}^{2}}$ for a homogeneous *k* − *coverage* network and $\frac{2k\pi R_{max}^{2}}{\left(\pi -\sqrt{3}\right)r_{min}^{2}}$ for heterogeneous *k* − *coverage* networks, where *r_min_* and *R_max_* are minimal sensing range and maximal communication range, respectively, in a heterogeneous network. The sufficient density to guarantee *k* − *coverage* for a homogeneous network is $\frac{2k}{\left(\pi -\sqrt{3}\right)r_{s}^{2}}$ and $\frac{2k}{\left(\pi -\sqrt{3}\right)r_{min}^{2}}$ for a heterogeneous network [36]. There are practical applications that try to find appropriate locations for sensors to maintain the network connectivity. For example, the work in [73] finds the optimal location for underwater sensors for the purpose of minimizing the transmission loss while maintaining the network connectivity; [74] finds the optimal location for reliable fault detection in a boiler system.

**Figure 7:**
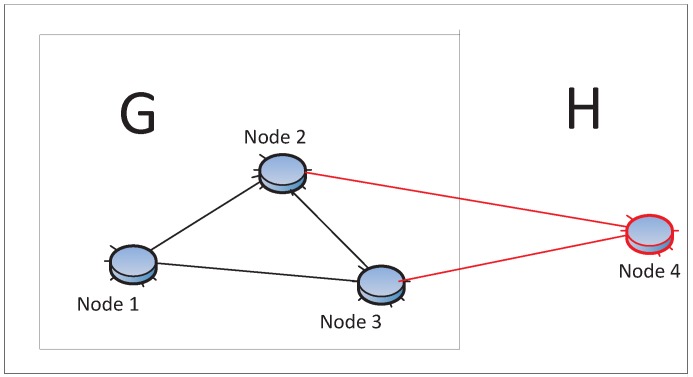
Construct a new 2 − *connected* network *H* from the existing 2 − *connected* network *G*.

(2) Construct New *k* − *Connected* Network from Existing *k* − *Connected* Network:

Let *G* be a *k−connected* graph; if *H* is a graph obtained from adding a new vertex and joining it to at least *k* vertices of *G*, *H* is also *k−connected* [14]. In Figure 7, the original *G* is a 2−*connected* network and the new graph *H* is constructed by adding the new Node 4. There are two disjoint links connected to *G* from Node 4, so *H* is a 2−*connected* network. Likewise, two disjoint *k −connected* components form a large *k−connected* component if there are *k−vertex* disjoint edges connecting them. There are several papers leveraging this theorem. This theory can also be used at the post-deployment stage; it will be discussed in Section 4.3. The work in [27] presents a distributed algorithm by locally constructing *k − connected* components and then joining two components by *k* disjoint edges between them to form one larger *k − connected* component. The work in [75] proposes an algorithm to construct a new fault-tolerant network by using some basic graphs. For instance, starting from really simple networks having 2, 3 and 5 nodes, it is able to construct a network that has cardinality *N* in the form 2^*i*^3^*j*^5^*k*^; the resulting graph will tolerate *i* + 2*j* + 2*k* − 1 faults.

(3) Spanning Tree:

The spanning tree is a tree that connects all vertexes in the graph. One graph may have more than one spanning tree, while the one has minimal total weight, called the minimal spanning tree (MST). Since it is a tree, it is a 1−*connected* network. Therefore, if one only emphasizes the 1−*connected* and minimal cost (e.g., energy), it is modeled as an MST problem, which can be solved by using the well-known Kruskal and Prim algorithm [76] and a distributed algorithm in [77]. MST can be solved with *O*(|*V*|) by a linear time randomized algorithm [78]. However, for *k* = 2, the problem of finding the minimal *k − connected* spanning sub-graph is NP-hard and has 1 + *ε* − *approximation* solutions [79]. There is no polynomial time approximation less than 1 + *ε* for any given positive constant *ε*. Let the number *d_MST_* be defined as the maximum possible degree of a minimum-degree MST spanning points from the space (e.g., if in Euclidean plane, *d_MST_* is five); the algorithm for 2−*connected* and 2−*edge*−*connected* is 2*d_MST_* − *approximation* [80].

### 4.3. Post-Deployment

After the nodes are deployed, topology control can be achieved by adjusting the transmission range (power) or scheduling the nodes’ status to be active or inactive. This ability is achievable in most sensor platforms, such as Crossbow and Sunspot. However, increasing the transmission range also causes higher signal inference. Consequently, it has a negative influence on link reliability.

#### 4.3.1. Connectivity, Energy-Efficiency and Coverage

In addition to connectivity, there are several other issues that should be taken into account, such as the lifetime of nodes. Higher transmission power results in faster energy consumption. For example, network connectivity is the largest when each node transmits at its maximum power, but it has the shortest lifetime. Extending the lifetime of the network in some scenarios is a desirable requirement, since the sensor nodes may be deployed in areas where humans are not allowed to enter, or the nodes cannot be replaced, or the battery cannot be recharged. Besides, if there are many redundant nodes, the coverage problem arises when scheduling some nodes to be active and others to be inactive, which is a common approach to maintain the network connectivity and coverage.

(1) Adjust Transmission Range:

The purpose is to assign each node a transmission power so that the resultant network is *k − connected*, and meanwhile, the transmission power is optimized. Optimizing energy while keeping network connectivity strong is a challenging task [8,34,81,82,83]. Most of the energy optimization and *k − connected* problems have been proven to be NP-complete [34]. Note that minimizing the total transmission power assignment is NP-hard even for a 1 − *connected* network [84,85]. There are centralized, distributed, or hybrid (e.g., [84]) approaches to address this problem.

If the node transmits with maximum transmission power and finds out the *k* optimal vertex disjoint path to each neighbor according to certain criteria (e.g., power cost), [86] proves that if each node maintains *k* optimal disjoint paths, the resultant network is *k − connected* globally, provided that all nodes use the maximum transmission power. In [84], the *k − connected* network is formed from three steps: first, the network becomes clustered; second, the cluster head assigns each node a communication power, such that each node in the cluster is *k − connected* (this is called the intra-cluster topology control); third, it is ensured that there are *k − disjoint* paths between adjacent clusters (this is called inter-cluster topology control). After these three steps, the resultant network is *k − connected* globally. The work in [87] aims at building up and maintaining *k − regular* and *k − connected* nodes by adding links between the overlay node. This approach forms groups consisting of *k* nodes, and each group is a *k − regular* network; it regards the group as a single node, then recursively using the same approach to form the final topology.

If the network already is a *k−connected* network, what is the condition preserving *k−connected* by changing the power based on the angle of neighbors? The work in [9] studies the angle of its neighbors. The cone of the degrees of nodes determines the connectivity. A node *u* transmits with the minimum power *P*, in order to ensure that in every cone of the degrees around *u*, there are some nodes that *u* can reach with power *P*; it is shown that taking angle $\frac{5\pi }{6}$ is a necessary and sufficient condition to guarantee that the network connectivity is preserved. The work in [9] tries to use fewer edges by removing edges, but preserves connectivity. Two basic mechanisms are adopted [9]: (1) start from the initial power, then increase the power until connected; and (2) start from the largest energy, then reduce it as long as the connectivity is not changed. The work in [81] preserves the connectivity of the network and meanwhile minimizes the transmission power. The work in [81] proves that the upper bound of the angle is $\alpha \leq \frac{2\pi }{3k}$. Note that many algorithms try to optimize the energy, inevitability incurring extra overhead.

(2) Schedule the Duty Cycle of Nodes:

Another common method to optimize energy is to schedule the duty cycle of nodes. There are usually three statuses in each sensor node: active, idle and sleep. The node in sleep or idle mode consumes less energy than the active mode. Nodes are scheduled to keep some nodes alive or active if they are on duty, while letting other nodes be in sleep mode in order to save the energy of the whole network, but still keeping the network connected. The work in [88] points out that the idle status consumes almost the same power as the active status, but the sleep model consumes much less than the active status.

For the case that every node is active with equal probability *p*, the asymptotic *k−coverage* results for a grid, random uniform and Poisson deployment are studied in [89]. The work in [56] utilizes random scheduling for the required coverage, but perhaps the active nodes are not connected; therefore, other nodes are turned on to guarantee connectivity if necessary. Node A is called the upstream node of B if A is the neighbor of B and one hop closer to the sink; accordingly, B is called the downstream node of A. The status of Node A is not only decided by the random scheduling assigned by the coverage scheduling, but is also determined by its downstream nodes. For example, Node A may be in sleep mode assigned by coverage scheduling, but it will be forced to switch to active mode if its downstream node is active and there is no path for this downstream node for transmitting data to the sink.

The model in game theory can be adapted to optimize the energy of WSNs, as well. For instance, the player in WSNs is the node. All nodes work in a non-cooperative manner. They selfishly conserve energy by refusing participants as relay nodes, but they can participant as a relay by offering some incentive to encourage cooperation. An incentive could be the token. The tax mechanism can be adopted, as well: a higher communication range introduces higher interferences to neighbor nodes; hence, they need to pay a high tax. The work in [90] surveys game approaches to formulate problems related to security and energy efficiency in WSNs.

#### 4.3.2. Link Quality

Increasing the network connectivity by power control is one way to improve the WSNs’ resilience, but the individual link quality is also important for resilient WSNs. Many power control approaches are employed to improve the link quality, thereby improving the reliability of the link. Higher transmission power helps to improve the data transmission reliability, but also introduces higher energy consumption and interference. There is a tradeoff among energy efficiency, link quality and interference.

There are papers devoted to conduct practical experiments to improve link quality, such as [38,91,92,93]. Adaptive and Robust Topology control (ART) [38], an adaptive topology control protocol, is based on extensive empirical study of the link quality in the indoor environment. It adapts the transmission power in response to variations in the link quality triggered by either environmental changes or varying degrees of network contention. ART monitors the outgoing packages and records the transmission failures during a defined time window. If the transmission failure is out of the defined threshold, ART increases or decreases the transmission power. One of the advantages is that ART does not always increase the transmission power to improve the link quality, because, in practice, it is not always true under high contention and interference. Instead, it may decrease the transmission power under high contention. The work in [91] proposes a protocol that applies a control-theoretic approach to control the packet reception ratio (PRR) directly. The receiver monitors all incoming packages and records the package transmission failures and then computes the transmission power needed for the next round. This information will be sent to the sender side. The work in [93] adjusts the transmission power by measuring the average received signal strength indicator (RSSI) and comparing with upper and lower thresholds to decide to increase or decrease the nodes’ transmission power. Adaptive transmission power control (ATPC) [92] employs a feedback-based transmission power control algorithm to dynamically maintain individual link quality over time. It makes proposals to the protocol with the idea of minimizing the power level by choosing the proper transmission power for each package transmitted, while guaranteeing the desired link quality. First, [92] reveals the relation between the transmission power and link quality indicator RSSI and link quality indicator (LQI), which is generally monotonic and continuous, but not deterministic. There are fluctuations in a small range at any transmission level. Based on empirical results, [92] formulates a predictive model to characterize the relation between transmission power and link quality. With this predictive model, the sender node adjusts the transmission power in order to guarantee a specific receiver the desired link quality. The work in [94] provides a localized configurable topology control (CTC) algorithm to achieve the desired path quality bounds in lossy WSNs.

#### 4.3.3. Connectivity Maintenance Protocols

There are many energy-aware routing protocols. In order to balance the connectivity and lifetime of the network, a good energy-aware protocol should: first, schedule as many nodes as possible to turn off; second, keep the network connected; third, offer as much total capacity as the original network; fourth, power saving should inter-operate correctly with whatever routing system [88]; and fifth, the algorithm needs to be distributed and highly efficient.

In a power saving technique for multi-hop ad hoc wireless networks called SPAN [88], a node volunteers to be a coordinator if it discovers that two of its neighbors cannot communicate with each other directly or through an existing coordinator. The Coverage Configuration Protocol (CCP) [15] protocol is a decentralized protocol that only depends on the local states of the sensing neighbors. CCP is able to guarantee both coverage and connectivity if the sensing range is less than half of the communication range, but if the sensing range is more than half of the communication range, CPP integrates with SPAN [88] to provide both coverage and connectivity. Probing Environment and Adaptive Sleeping (PEAS) [95] schedules a required number of nodes as awake by adjusting the sleep period in the presence of failures. It sends the probing messages to the nodes within the probing range, which is a value lower than the communication range. Any nodes within its probing range will send back a reply message. The sleeping nodes keep working only if they do not receive the reply messages. In this way, it keeps the network connected. The work in [27] presents a greedy algorithm: it starts from a complete graph, then reduce nodes to reach the desirable connectivity, or starts from an empty graph and later connects edges until the desirable connectivity is reached; then, it cuts nodes, but maintains the same connectivity. The second step is to reduce the redundant nodes.

### 4.4. Re-Deployment

The last sections assume that deployed WSNs nodes cannot get in touch, or relocate, or add nodes once they are deployed. WSNs can adapt to keep connectivity. However, for real WSN node deployment, it is still possible that nodes are able to be added or relocated in some circumstances, either by human beings or robot-like devices carrying new nodes to the field. The goal of the node placement is to achieve some desired performances, such as energy optimization, maintaining or improving connectivity by the lowest number of nodes. Adding new nodes or edges set to make a graph being *k − connected* is also called the connectivity augmentation problem. The boundaries are given in [96]: $\frac{2n}{3}$ additional edges are required in some cases, and $\frac{6n}{7}$ additional edges are always sufficient, where *n* is the total number of sensor nodes. The connectivity augmentations can be done by populating new nodes to achieve a particular architecture that has strong connectivity. For instance, the goal of a new node may be constructing a complete network where each node connects to all other nodes or a regular graph where each node has the same number of neighbors, or circulants, like Harary graphs.

The work in [3] categorizes the placement strategies into static and dynamic depending on whether the optimization is performed at the time of deployment or while the network is operational, respectively. The behavior of mobile nodes can be the synchronous, asynchronous or semi-synchronous model [97]. In the synchronous model, all relay nodes take action at the same time; in the asynchronous model, all relay nodes act individually; in the semi-synchronous model, a subset of nodes moves, but not all.

#### 4.4.1. Integer Linear Programming

The node placement problems can be formalized as a linear programming (LP) problem or an integer linear programming (ILP) problem. ILP could be an optimal approach to find the optimal numbers or positions of new nodes to improve or maintain connectivity if the network is small, but for a large network, the LP or ILP are not applicable.

For instance, the energy optimization for *k − connected* networks can be regard as an LP problem. The constraints and objective functions are given in [35]. The work in [98] formulates the optimal number of relay nodes to guarantee connectivity and coverage to be an ILP problem. The work in [99] investigates the node placement strategies to achieve five objectives: mean overall residual energy, mean minimum residual energy, mean number of nodes over the threshold, mean overall traveled distance and mean maximum distance. The problem is formalized to be a ILP, but unfortunately, it has a huge number of constrains, including the positions of all deployed nodes and their status information.

The work in [100] proposes an ILP method to solve minimal number of relay node placement issues related to the energy consideration to guarantee network connectivity. There are 20 constraints and many variables depending on the number of relay node positions. It shows that for *k* small (one or two) and a network that is not that big (tens of nodes), the problem is solvable within a reasonable time. However, if the network is large, and for a bigger *k* value, it seems that it is still intractable with ILP. Therefore, few problems can be solved in polynomial time. In turn, most of them are very difficult to solve with the optimal solution. If the original problem is NP-complete, the corresponding LP or ILP is also NP-complete.

#### 4.4.2. Steiner Tree

Given a weighted undirected graph and a subset of terminal nodes, finding a minimum cost tree spanning terminals is called the Steiner tree problem, which is NP-hard. The difference between the Steiner tree problem and the MST problem is that the Steiner tree allows one to construct or to select intermediate connection points to reduce the cost of the tree. Those intermediate nodes are the optimal new nodes that the network needs to maintain the connectivity and low cost. For now, the best algorithm has 1.39 − *approximation* for a weighted Steiner tree [101]. MST can be used to solve the Steiner tree, but it is not necessary for it to be an optimal solution. Placing the minimum number of nodes for maintaining network connectivity, such that the transmission range of each sensor does not exceed a threshold, is the minimum number of Steiner points (SMT-MSP) [102], which is NP-Hard, as well. Many node placement optimization problems can be reduced to an SMT-MSP problem, e.g., [44,45,97,103,104].

#### 4.4.3. Heuristic Algorithms

Node optimal placement is a very challenging problem. The work in [97] states that no local algorithm can achieve good approximation factors for the number of relays. Many of them have been proven to be NP-complete or NP-hard, even when only wanting to achieve a 2 − *connected* network [105,106].

Since most relay node optimizations are NP-complete or NP-hard problems, many heuristic solutions have been proposed. The work in [104] first uses a minimal spanning tree algorithm over points set *P*, then inserts new points for those edges longer than *R*; the resultant tree has no edges longer than *R*. This algorithm has 5 − *approximation*. The work in [102] improves the approximation of [104] and proposes two algorithms that have a performance approximation of three and 2.5, respectively. Note that they simply consider the 1 − *connected*, since it is a tree. Similarly, in heterogeneous networks with the nodes with distinct transmission ranges, the node placement for full *k − connected* (considering all nodes) and partial *k − connected* (not considering the populated nodes) is an NP-hard problem [28]. The algorithms proposed in [28] have two phases: (1) to solve the minimum *k − connected* spanning graph for the initial network; and (2) to put the necessary relay node calculated graph in the first phase. According to the first phase, it’ is not difficult to know where and how many relay nodes are needed. However, those methods need to know the location of each node.

The work in [105] proposes the *O*(*D* log *n*) − *approximation* replacement algorithm of the minimal number of relay nodes for a 2 − *connected* network, where *D* is *D*(1, 2) − *diameter*. It assumes that regular nodes have the same communication range, but the relay nodes have double the range of other nodes. The work in [97] tries to maintain the network as connected while minimizing the number of relay nodes. It considers the global and local algorithms. Notice that there are two kinds of nodes: terminal and relay nodes. The difference is that the terminal nodes are stationary, and the range is one, while relay nodes can move and have a range of more than one. The work in [107] proposes a relay node placement algorithm for a *k − connected* (not only 2−*connected*) network based on the particle swarm algorithm, also considering the heterogeneous feature of WSNs where nodes have different communication ranges. Notice that the algorithm runs off-line. The work in [107] considers that heterogeneous WSNs are only for original nodes, for all new joined relay nodes have the same communication range. The work in [108] proves that the algorithms have complexity *O*(*n*^6^) or *O*(*cn*^3^) for any *k*. The work in [109] proposes two distributed relay node positioning approaches, based on virtual force-based movements and game theory, respectively, to guarantee network recovery by minimizing the movement cost of the relay nodes. The work in [110] aims to position the minimum number of additional relay nodes by the branch-and-cut-based algorithm, such that signals from sensor nodes communicate to the base station within a pre-specified delay bound.

## 5. Multi-Path Routing Protocol

Once the sensor nodes are deployed in the area of interest using the approach in Section 4, the network will be *k − connected* from the topology point of view. The routing protocols are used to find a specific path from the source to the destination according to specific routing algorithms. When the nodes or links fail on this path for whatever reason, the routing protocols need to detect failures first and then find alternative paths to the destination. As a consequence, the routing protocols play an important role in resilient WSNs.

The work in [111] classifies the routing protocols in wireless *ad hoc* and mesh networks into different categories: proactive, reactive, geographical, multi-path, hierarchical, flow-oriented, Wireless Mesh Network (WMN) and multicast, power aware, *etc*. Of course, some of them share common properties. However, surveying routing protocols in WSNs is out of the scope of this paper; the reader is referred to [111] for more details. Multi-path routing and energy-aware protocols, such as [112], Robust and Energy Efficient multipath Routing (REER) [113], Energy Efficient Multipath Routing protocol for WSNs (referred as RELAX) [114], Low-Interference Energy-efficient Multipath ROuting (LIEMRO) [115], Energy Efficient Fault Tolerant Multipath (EEFTM) [116] and Probing Environment and Adaptive Sleeping (PEAS) [95], are suitable for resilient WSNs.

The challenges here are how to design low overhead and low complexity routing algorithms, taking into account heterogeneous or homogenous network models, stationary or mobile networks, using 2D or 3D models, knowing the locations of nodes (e.g., by GPS) or not, localized (distributed) or centralized (globalized) algorithms, *etc*.

### 5.1. Complete Disjointed and Brained Path

There are two popular routing protocols, Dynamic Source Routing (DSR) [118] and Ad-hoc On-demand Distance Vector (AODV) [117] in WSNs. DSR keeps multiple paths in the routing table for increasing reliability. This gives DSR good fault tolerance. AODV uses multi-round discovery, exploring alternative paths to establish a route. However, both DSR and AODV are on-demand routing protocols, which may lead to significant latency and overhead at every path-finding stage. Multi-path routing eliminates the routing discovery process unless there is no path available.

**Figure 8:**
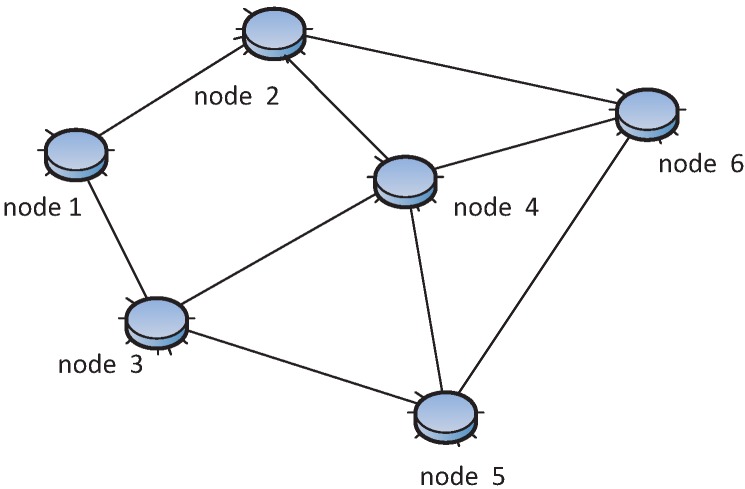
Disjoint paths and brained paths. (1, 2, 6) and (1, 3, 5, 6) are disjoint-paths. (1, 3, 4, 6) and (1, 3, 5, 6) are brained paths.

In general, there are two kinds of multi-path routing, as illustrated in Figure 8: (1) complete disjointed path: all paths do not share common nodes except the source and destination nodes. There is much research on this topic. The algorithm to find *k − disjoint* paths earlier can be found in [119,120,121,122]. The second is (2) brained paths: the paths are not completely disjoined; there are many partially-disjoint alternate paths. There is a tradeoff between resilience and maintaining overhead [47]. For comparable resilience to patterned failures, the brained multi-path expends only 33% the energy of complete disjoint paths and has 50% higher resilience to isolated failures [8].

Formally, there are two ways to evaluate the relation between two paths: disjointness and stretch [122]:

(4) $$disjointness=1-\frac{|P_{primary}\cap P_{backup}|}{|P_{primary}|}$$

(5) $$stretch=|\frac{P_{backup}}{P_{primary}}|$$

*P_primary_* and *P_backup_* are defined as the set of links in the primary and the backup path. The resilient network needs *disjointness* close to one and a stretch as small as possible. Paths must be chosen to achieve the highest reliability possible, however. The work in [123] proves that in order to improve the reliability of multi-path routing, if the primary path is the shortest path, then the second path should be as short as possible; while if the primary path is not the shortest path, then the second path should differ from the first as much as possible.

### 5.2. Multi-Path Searching Algorithm

If it is only required to confirm that the network is 1 − *connected* or not, one can simply run the depth-first search or breadth-first search, both having *O*(|*V*| + |*E*|). The work in [124] employs the breadth-first search algorithm to judge whether a node caused the network partitions. In this case, the link rebuilding strategy will start to achieve the topology connectivity. The work in [125] proposes an optimal algorithm to find the single-source shortest non-negative path with complexity *O*(|*E*| + |*V*| log |*V*|). The finding the maximum number of *k − disjoint* path problem can be solved by network flow theory, specifically the minimum cut or maximum flow theory. There are many polynomial time algorithms solving maximum flow problems. So far, the best algorithm has complexity $O(|V||E|\log\frac{{\left|V\right|}^{2}}{\left|E\right|})$ [96]. The multi-path from the source and destination for all nodes can be found in *O*(*kN*^2^) running the time algorithm [34].

However, for the WSNs, there are always some special considerations, such as the computational capacity, that need to be considered before implementing the algorithm, so an *O*(*kN*^2^) running time perhaps is unacceptable in the WSNs. Hence, the problems that have been solved in graph theory may be either hard or even impossible to apply to WSNs, because they have an unacceptable algorithm complexity. Therefore, the heuristic algorithms are more feasible. For example, a greedy algorithm can work as follows: first, the algorithm runs to choose one path to the destination by selecting the best hop at each step, then running the same algorithm again to find the second path except for the node (link) that has already been chosen, and so on, such as routing protocols [8,126,127].

**Figure 9:**
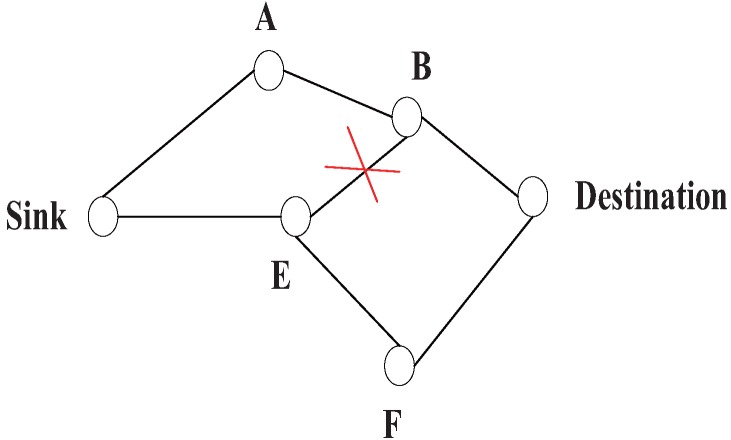
Construct disjoint paths.

The work in [8] realizes a multi-path routing algorithm by means of only using the local information. The sink first floods data to the destination according to the metric, like lowest delay or loss. In the end, the primary path is established, as the path (Sink-A-B-Destination) in Figure 9. After that, the destination sends the “alternative path reinforcement” to the sink by choosing the second preferred neighbor, and along the way back to the source, each neighbor chooses the second preferred neighbor that has not been chosen before. If it has been chosen, it has to choose another one. As illustrated in Figure 9, the path (Destination-B) has been chosen in the primary path, so at this step it will not choose (Destination-B) as part of the secondary path. Instead, it will select (Destination-F-E-Sink) as the secondary path. Finally, the 2 − *disjoint* path is formed. Note that: (1) this method only uses the local information; and (2) during the time used in setting up the network to find the multi-path, it might take a somewhat long time and consume too much energy.

The multi-path algorithms are preferably distributed, e.g., [128,129]. The work in [128] is a distributed algorithm to find out a complete disjoined path. It is based on any other existing routing protocols to find the multi-path from the source to the destination and then proposes a distributed algorithm to refine those paths in order to eliminate the paths that have mutual interference. The work in [129] proposes a new routing protocol called GRAd, which is a routing protocol that makes no attempt to identify which of its neighbors are delivering a packet. It considers the mobility of sensor nodes. The nodes travel towards randomly chosen locations at random speeds and pause for a fixed amount of time after reaching the destination. The simulation results show that GRAd is robust in the face of a changing topology, because it enlists multiple neighbor nodes to relay messages from one place to another. Even if one node moves out of place, other nodes are often available to deliver the packet without resorting to rebuilding the route. However, a negative consequence of this high robustness as [129] shows is that the routing load is high. The work in [26] proposes resilient routing reconfiguration (R3), which consists of an off-line pre-computation phase and an online reconfiguration phase. By converting topology uncertainty into uncertainty in rerouting traffic, it applies linear programming duality to find the optimal route and protection route.

## 6. NP-Complete and NP-Hard Problems in Resilient WSNs

As mentioned in previous sections of this paper, many problems in WSNs with regard to resilient WSNs turn out to be NP-complete or NP-hard, which is summarized in this section. For NP-complete and NP-hard problems [14], there is no polynomial-time algorithm to solve them. However, there are still some NP-complete and NP-hard problems being provably bound on the solution, called the *α − ratio* or *α − approximation* algorithm. The *α − approximation* algorithm is a polynomial-time algorithm whose solution cost is at most *α* times the optimal solution cost. On the other hand, many related works have been focused on providing heuristics. However, heuristics could have arbitrarily poor performance and do not guarantee provable running time, like the *α − approximation* algorithm.

If the problems are NP-complete or NP-hard, usually a heuristic algorithm is proposed; then, its correctness is proven, and the *α − approximation* of its performance is presented if it exists. If the algorithm needs to know the status of the entire network or to have a physical (or logical) control center, it is called a centralized algorithm; otherwise, it is a distributed algorithm. Table 1 lists many problems and algorithms regarding resilient WSNs. Note that most of the algorithms proposed are centralized.

**Table 1:** NP-complete and NP-hard problems in resilient WSNs.

Ref.^1^	Problems	*α* − Ratio	Complexity	Distr.^2^
[35]	Minimize power while maintaining k-fault tolerance	*O*(*k*) *O*(*k* log *k*)	*NA*^3^	Yes
[81]	Find k-connectivity sub-graph with minimum cost	*NA*	*NA*	Yes
[121]	Find *k* pairwise vertex or edge disjoint paths in an acyclic directed network with length bounded by an integer; *L* is the sum of all edges	*NA*	*O*(|*V*|^*k*+1^*L*^2*k*−2^) *O*(|*V*|^*k*+1^*L^k^*)	NO
[121]	Find two vertex or edge disjoint paths in a directed network, such that the maximum length of two path is minimized	at least 2	NA	NA
[123]	The reliability of the two disjoint-path connection equals 1	NA	*O*(|*E*| log |*V*|)	NO
[76]	Assign minimized transmission power to each sensor, such that the induced topology containing only bidirectional links is strongly connected	*k*^2^ + *O*(*k*)	NA	NO
[27]	Establish k-connectivity by placing additional sensors geographically between existing pairs of sensors with a minimum number of additional sensors	2	*O*(|*V*|^2^ log *V*)	NO
[102]	Place the minimum number of relay nodes for maintaining network connectivity, such that the transmission range of each sensor does not exceed a constant	3, 2.5	*O*(|*V*|^3^)	NO
[104]	Steiner tree problem with minimum number of Steiner points and bounded edge length (STP-MSPBEL)	5	NA	NO
[80,105]	Minimal number of relay nodes for a 2-connected network	*O*(*D* log *n*) 10	*O*((*k*|*V*|)^2^) [80]	NO
[79]	Find the smallest 2-connectivityweighted spanning sub-graph	1 + *ε*	NA	NO
[45,103]	Find the minimum count and position of relay nodes	NA	*O*(*n*^4^) [103]	NO
[106]	Minimum number of edges required to be added to obtain a 2-vertex or 2-edge connected plane geometric graph	NA	NA	NO
[28]	A minimum number of node placement for full k-connectivity and partial k-connectivity in the heterogeneous networks in terms of non-identical transmission range	*O*(*σk*^2^) *O*(*σk*^3^)	NA	NO
[85]	Directed k-strong connectivity with a minimal overall power assignment for uniformly-spaced nodes on a line or planar space	$min{\left(2,\frac{\Delta }{\delta }\right)}^{\alpha }$ *O*(*k*^2^)	NA	NO

^1^ Reference; ^2^ Distributed algorithm; ^3^ Not applicable.

## 7. Open Issues

In this section, several challenging issues are discussed and expected to be considered in future research.

(1) More Realistic Transmission Model for WSNs:

The network model in many research papers is still too ideal. As shown in [38] for a TelosB testbed and [92,93] for a MicaZ testbed, the empirical studies performed show that link quality varies significantly over time, due to human activity and the multi-path effect. The work in [38] suggests that increasing transmission power up to −7 dBm can improve the link quality; however, by further increasing the transmission power, the link quality actually decreases. For a specific transmission power, communication distance and antenna direction, the link quality still varies over time [92]. Those practical results imply that the realistic model of network is much more complex. The probabilistic approach can play a role here. The work in [130] provides one alternative model for an irregular communication range. The irregular radio is modeled as the degree of irregularity (DOI), which is defined as the maximum radio range variation per degree change in the direction of radio propagation. There are upper and lower bounds for radio propagation. If the neighbor node is out of the upper bound, then it is out of the communication range; while if the node is below the lower bound, it is in the communication range; if the distance is between the upper and lower bounds, the model is described as DOI. In other words, the DOI parameter can be changed to approximate a specific type of hardware (e.g., MicaZ) or environment (e.g., indoors). Our latest research proposes using the Cox process to model the irregularity of the communication range [40].

In short, the transmission model, such as the disk, is inappropriate to model the WSN nodes. The probabilistic approach can be introduced to get more accurate transmission models.

(2) Sink Node:

Most research studies the connectivity issues assuming that all nodes in the network are equally important, without considering the role of sink or base station nodes, which are the data centers that all sensors data take as the final destination. To some extent, if the sink node losses connection with others, the rest of the nodes in the network could be useless for applications. Therefore, a network model that considers the role of sink node is necessary.

(3) Higher Dimension Model:

2−*dimensions* is a commonly-used model for WSNs. However, WSNs can apply to 3−*dimensions*, such as underwater, atmospheric, high buildings with many floors, mountains or space communications. Those models, especially referring to the transmission model, are necessary to check their performances in 3 − *dimensions*. Very few researches consider resilient WSNs issues in 3 − *dimensions*. The conclusion in [81] can be applied from 2−*dimensions* to 3−*dimensions* when *k* < 3. The transmission range must be at least 1.7889-times the sensing range in order to maintain connectivity among nodes for the truncated octahedral deployment, while the transmission range is between 1.4142- and 1.7889-times the sensing range for the hexagonal prism placement strategy or the rhombic dodecahedron placement [131]. The work in [132] proposes how to construct the full coverage and *k − connectivity* network in 3 − *dimensions*, where *k* ≤ 4. Nevertheless, more proposals on higher dimensions are still imperative. A more complete survey on the topology control based on a 3−*dimension* model for WSNs can be found in [133].

(4) Mobility:

WSNs may be a highly dynamical network where the nodes move frequently, for example the WSNs can be applied to vehicle monitoring or wearable sensor devices. It is interesting to study the network connectivity in a dynamical network. It is very likely that those problems, which are intractable in static networks, could not be easily solved in dynamical networks either. Despite connectivity being more difficult to model and maintain in a mobile environment, the network resilience may benefit from nodes’ mobility. For example, carefully controlling the node trajectory may enhance connectivity or WSNs can recover from failures more quickly.

(5) Distributed Algorithms:

Unfortunately, as shown in Table 1, many topology control approaches in graphs are centralized and have unaffordable complexity for WSNs. The WSNs are large distributed systems, so heuristic algorithms have to be distributed, as well. Those non-distributed algorithms harm the scalability and require higher maintenance cost, so implementing the algorithms in a distributed manner needs to be further studied.

## 8. Conclusions

This paper surveys resilience issues in WSNs from the topology control and multi-path routing point of view. In general, the key to keeping the network resilient against failures is to maintain the network as *k − connected*, where *k* is the most important indicator for resilience. The conclusions in this survey are the following:

(1) The first step to study the resilience issue is to employ the network models and failure models. This work shows that the network model is quite complicated. To fully capture the most important features of sensor nodes, one should adopt more realistic models and avoid using ideal models. We believe that the most commonly-used models, such as a homogeneous network and a regular radio model, are inappropriate to model WSNs.

(2) In order to improve the resilience of WSNs, many approaches can act on three different deployment stages to improve the connectivity. At the pre-deployment stage, in the case that the nodes are randomly deployed, the theoretical result shows what is the condition to have the network *k − connected* with high probability; in the case that the node location can be controlled, the required *k − connected* network can be constructed. At the post-deployment stage, the main methods are controlling the transmission range (power) or scheduling the node status to achieve the desired connectivity or link quality. In the re-deployment stage, WSNs can maintain or improve connectivity by placing new relay nodes. By doing this, this paper provides a framework that can help researchers design resilient WSNs at different stages.

(3) Once the network is deployed, multi-path routing can be used to establish resilient paths from the source to the destination.

(4) It turns out that many issues related to WSNs’ resilience are very challenging; they are either NP-complete or NP-hard problems. Therefore, optimal solutions do not exist. This paper identifies those problems and points out that those most proposed algorithms are still centralized. It is expected that more distributed heuristic algorithms will be proposed in the future.

(5) In most cases, the *k −connected* network is not the only goal desired; other goals, such as energy efficiency, coverage and the number of nodes deployed, are also need to be taken into account when designing resilient WSNs.

(6) Considering the 3 − *dimensions* and mobile network model in the study of resilient WSNs may be interesting.

(7) Although the 1−*connected* topologies are not able to tolerate node failures (such as chain topology and tree-based topologies), one can construct a fault-tolerant network, e.g., *k − connected* network (*k* > 1), by using many 1 − *connected* networks.

(8) Finally, this paper found that a great part of the literature is dedicated to solving the *k−connected* problem for only one deployment stage. It is possible and necessary that those approaches at different deployment stages can be integrated to maximize the fault tolerance capability. For instance, at the pre-deployment stage, the initial network should be built to be *k − connected* with high *k*; at the post-deployment stage, the transmission range to maintain a *k−connected* network should be controlled; if node failures occur so that it is impossible to maintain a network *k − connected*, the approaches that calculate the locations for new nodes to recover connectivity can be used.

This survey intends to provide researchers the state-of-the-art solutions concerning resiliency issues in WSNs at different design stages. A novel perspective on designing resilient WSNs is provided, as well. In order to do this, we clarified the concept of resilience and offered clear guidelines on the appropriate models that should be considered, as well as in which stage(s) the techniques can be applied, and we identified the problems that are still very challenging. Thus, this paper can serve as a guideline for the design of resilient WSNs.

Currently, we are developing a real sensor network using our proposed topology control systems. We deployed about 10 sensor nodes in indoor and outdoor environments. The self-adaptive control system, which aims at making a tradeoff between network connectivity and energy consumption, runs in each sensor node. The preliminary results showed that both the network resilience and energy consumption can be improved. When few nodes fail, the average number of nodes and the proportion of nodes that can be connected to the BS are relatively stable, which implies the network’s stability in the presence of node failures.
